# Supplementary material for: Identification of photoperiod-induced specific miRNAs in the adrenal glands of Sunite sheep (Ovis aries)
Source: Front Vet Sci. 2022 Jul 22;9:888207. doi: 10.3389/fvets.2022.888207 (PMC9354845; doi:10.3389/fvets.2022.888207)
Supplement: Supplementary file 1 [file Data_Sheet_1.ZIP › Supplementary file/miRDeep2 file.zip!/miRDeep2 file/Novel_Structure/LP42A1/Novel_245.pdf]

| | | |
|---|---|---|
| Provisional ID | : | NC_019460.2_8691 |
| Score total | : | 166.4 |
| Score for star read(s) | : | 3.9 |
| Score for read counts | : | 159.2 |
| Score for mfe | : | 1.7 |
| Score for randfold | : | 1.6 |
| Score for cons. seed | : | |
| Total read count | : | 324 |
| Mature read count | : | 302 |
| Loop read count | : | 0 |
| Star read count | : | 22 |

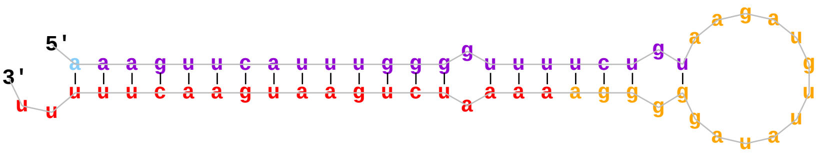


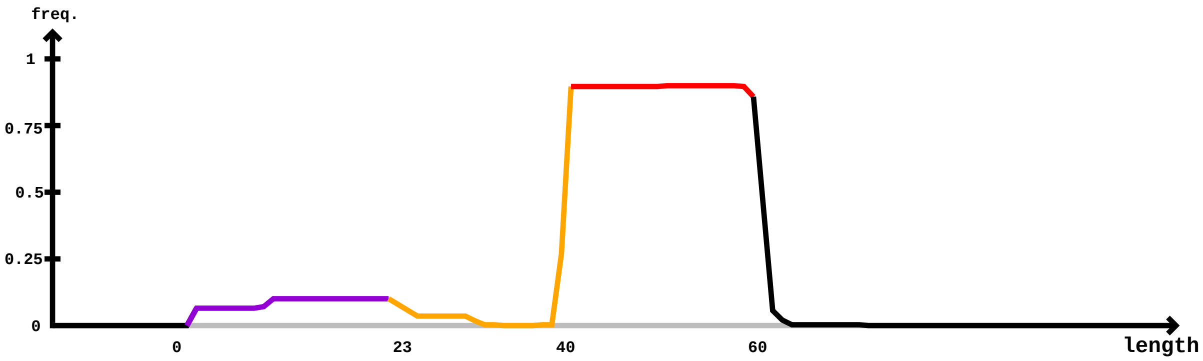


Star Mature

```
5'- uggguugacccaaaaguucauuugggguuuucuguaagauguuauagggggaaaaaucugaaugaacuuuuuggcuaacccaaaguuuacuuauuuauuuagguuagcuacuuaa   -3'   obs
    uggguugacccaaaaguucauuugggguuuucuguaagauguuauagggggaaaaaucugaaugaacuuuuuggcuaacccaaaguuuacuuauuuauuuagguuagcuacuuaa         exp
    ((((((.((.((((((((((((((((.((((((.(............).)))))).))))))))))))))).)).))))))).((((.(((((......))))).))))......   reads   mm   sample
    .............aaguucauuugggCuuuucugu................................................................................   21      1    seq
    .............aaguucauuugggguuuucugu................................................................................   1       0    seq
    ....................uuugggguuuucuguaagaugu.........................................................................   1       0    seq
    ....................uuugggguuuucuguaagauguuau......................................................................   1       0    seq
    .....................uugggguuuucuguaagaugu.........................................................................   5       0    seq
    .....................uugggguuuucuguaagauguu........................................................................   5       0    seq
    .................................................Agaaaaaucugaaugaacuuu.............................................   1       1    seq
    ...................................................aaaaaCcugaaugaacuuuu............................................   4       1    seq
    ...................................................aaaaaucugaaugaacuuuuA...........................................   1       1    seq
    ...................................................aaaaaucugaaugaCcuuuuu...........................................   1       1    seq
    ...................................................aaaaaucCgaaugaacuuuuu...........................................   1       1    seq
    ...................................................aaaaaCcugaaugaacuuuuu...........................................   15      1    seq
    ...................................................aaaaaucugaaugaacuuuuu...........................................   66      0    seq
    ...................................................aaaaaucugaaugaacuuuuuUg.........................................   2       1    seq
    ....................................................aaaaucugaaugaacuuuu............................................   9       0    seq
    ....................................................aaaaucugaaugaacuuuuA...........................................   6       1    seq
    ....................................................aaaCucugaaugaacuuuuu...........................................   3       1    seq
    ....................................................aaaaucugaaugaacuuuuu...........................................   126     0    seq
    ....................................................aaaaucugaaCgaacuuuuu...........................................   8       1    seq
    ....................................................aaaaucugaauAaacuuuuu...........................................   1       1    seq
    ....................................................aaaaucAgaaugaacuuuuu...........................................   1       1    seq
    ....................................................aaaaucugaaugaacuuuuuU..........................................   6       1    seq
    ....................................................aaaaucugaaugaacuuuuug..........................................   36      0    seq
    ....................................................aaaaucugaaCgaacuuuuugg.........................................   2       1    seq
    ....................................................aaaCucugaaugaacuuuuugg.........................................   6       1    seq
    ....................................................aaaaucugaaugaacCuuuugg.........................................   1       1    seq
    ....................................................aaaaAcugaaugaacuuuuugg.........................................   1       1    seq
    ....................................................aaaCucugaaugaacuuuuuggc........................................   2       1    seq
    ....................................................aaaaucugaaugaacuuuuuggc........................................   4       0    seq
    ..............................................................ugaacuuuuuggcuaacccaa................................   1       0    seq
```
